# The Story of the Insane from Year to Year

**Published:** 1896-10-24

**Authors:** 


### THE STORY OF THE INSANE FROM YEAR TO YEAR.

Ninth Series.

### SOUTH AUSTRALIAN HOSPITALS FOR THE INSANE.

This report embodies statistics of the asylums at Parkside and Adelaide, from which it would seem that these hospitals contained 930 patients at the close of the year 1895. There were 212 admissions, 54 recoveries, 48 discharged relieved, and 73 deaths. The percentage of discharges stands at 481. on the admissions, and the deaths at 8·4 on the average number resident. Table 11, which gives the assigned causes of the insanity in the year's admissions, is almost useless, as 112 are put down as unknown, and 10 owing to drink, and only 1 to hereditary influence which is, of course, sheer nonsense. Of the deaths, 49 were owing to cerebral or spinal diseases, and 3 to consumption. Dr. Paterson, being absent on leave, the report is written by Dr. Cleland, who devotes considerable space to the discussion of the real or supposed increase of insanity, and he arrives at the very comforting conclusion that the "occurrence of insanity is on the decrease in the general population of Southern Australia, and that the population is considerably less insane than it was nineteen years ago." It would further seem from Table 3 that while in England the ratio of persons of unsound mind is 3·09 per thousand, it is only 2·58 in South Australia. The average cost per head per week is about 9s. 4d.

### NORWICH CITY ASYLUM.

An increase from 304 to 313 has to be chronicled at this asylum. There were 89 first admissions, 21 readmissions, 35 recoveries, 29 relieved, and 34 deaths. The percentage of recoveries on admissions stands at 34·31, and that of the deaths at 11·22 on the average number resident. Of the deaths 22 were caused by cerebral and spinal diseases, 4 by consumption, and 1 by diarrhœa. Fifteen of the year's admissions were driven mad by intemperance in drink and 13 by hereditary influences; 15 by congenital defect and 24 by moral causes. Dr. Harris bestows on his subordinate staff a well-earned meed of praise for conscientious discharge of duty, and it is always a pleasure to notice this feeling existing between the head of an asylum and the attendants. We also commend Dr. Harris's views of what cricket should be in asylums, and we regret that his ideas on the subject of games are not more commonly held. The storm which visited England in the spring of 1895 affected the asylum very seriously, for over 4,000 slates were blown off the roofs and 400 panes of glass broken. It is satisfactory to learn that the Norwich patients are not compelled to keep the Puritan Sunday, for on the evening of the storm, which was a Sunday, a concert was held in the hall and recitations given. The visitors record their "unabated confidence" in Dr. Harris. The average weekly cost per head was 8s. 10¼d.

### IPSWICH BOROUGH ASYLUM.

The numbers fell during year from 264 to 259. There were 85 first admissions, 13 readmissions, 44 discharged recovered, 14 relieved, and 29 deaths. The recoveries reached the creditable percentage of 44·8 on the admissions, and the deaths were 11 per cent. on the average number resident; the latter, like the former, being above the average in our public asylums. Fourteen of the deaths were caused by cerebral and spinal diseases, and 7 by consumption. Drink accounts 6 of the admissions, and hereditary influence for 17. Scarlet fever broke out in the asylum, but was fortunately limited to the first two cases infected. The asylum is to be enlarged by providing increased dormitory accommodation on the male side. The average cost per head per week is a little under 9s. 2d.

